# Case report: exercise-associated sudden death in a Thoroughbred racehorse with cardiac lesions

**DOI:** 10.1007/s11259-026-11384-0

**Published:** 2026-07-04

**Authors:** Hernando Daniel Acevedo, Francisco Uzal, Jeff Blea, Eileen Henderson, Akinyi Nyaoke, Carlos Omar Schild, Javier Asin

**Affiliations:** 1 California Animal Health and Food Safety Laboratory System, San Bernardino branch, University of California–Davis, San Bernardino, CA USA; 2 School of Veterinary Medicine, University of California, Davis, CA USA

**Keywords:** Cardiac examination protocol, Exercise, Horse, Sudden death, Thoroughbred

## Abstract

Exercise-associated sudden death (EASD), defined as acute collapse and death in an apparently healthy racehorse during or within ~1 h after exercise, is currently among the top concerns in the racing industry. Cases of EASD with no cause of death identified at necropsy are frequently presumed to be of cardiac origin. In such cases, in addition to a regular necropsy, a cardiac examination protocol is performed that includes a detailed gross examination and microscopic evaluation of 11 regions of the heart. Very often this protocol is unrewarding because there are no microscopic lesions to explain the presumed cardiac event. Here we describe the necropsy of a 3-year-old, Thoroughbred gelding that collapsed a few minutes after a workout. The heart was grossly unremarkable, but significant histologic lesions that included multifocal to diffuse cardiomyocyte degeneration and necrosis, lymphoplasmacytic myocarditis, and myocardial fibroplasia were found, particularly in the right and left ventricular free walls and in the interventricular septum. Other lesions included severe pulmonary edema and congestion, splenic congestion, an open castration wound with localized suppurative inflammation and bacteria in the scrotum, and lymphoplasmacytic interstitial nephritis. We believe that the cardiac lesions were severe and extensive enough to have contributed to death. A cause for such lesions was not definitively identified, but it is possible that the infected castration wound predisposed to myocarditis. This case serves as an example of the use of the cardiac examination protocol to map and evaluate lesions in EASD cases.

**Supplementary Information:**

The online version contains supplementary material available at https://doi.org/10.1007/s11259-026-11384-0.

## Background

Sudden death (SD) is defined as an unexpected death in a closely observed and apparently healthy horse with no prior clinical signs noted (Lucke 1987; Uzal et al. 2026). In equine athletes, exercise-associated sudden death (EASD) is defined as a case of SD that occurs during exercise or within ~ 1-hour post-exercise (Diab et al. 2017; Uzal et al. 2026). Cases of EASD may or may not have significant cardiac lesions. In many cases, despite the lack of significant cardiac lesions, death is presumed to be of cardiac origin based on the lack of non-cardiac abnormalities that could directly lead to SD (Diab et al. 2017; Lyle et al. 2011; Uzal et al. 2026). Many studies have been performed to try to identify the etiology of EASD in racehorses, and some causes include exercise-associated fatal pulmonary hemorrhage, massive thoracic and/or abdominal bleeding due to vascular ruptures, cardiac tamponade as a result of aortic rupture, skull or cervical vertebral fractures, and myocardial fibrosis or other cardiomyopathies (Boden et al. 2006; Buergelt 2003; Gelberg et al. 1985; Gunson et al. 1988; Johnson et al. 1994; Kiryu et al. 1987, 1999; Lyle et al. 2011; Platt 1982; Uzal et al. 2026). Nevertheless, in most cases of EASD no gross or microscopic lesions other than pulmonary edema, congestion and hemorrhages are identified, and the cases are frequently labelled as “necropsy-negative” (Diab et al. 2017).

Several cardiac lesions can occur in horses; lesions can be congenital or acquired, and may occur in the pericardium, myocardium, endocardium, and/or the valves (Buergelt 2003; Uzal et al. 2026). Some studies report 4–11% of cardiac lesions with a suspected or confirmed significant role in equine EASD cases (Lyle et al. 2011; DeLay 2017; Molesan et al. 2019; Hellings et al. 2022; Nath et al. 2022), including chamber dilation, valvular changes, right atrial hypertrophy and right ventricular dilation, left ventricular hypertrophy and aortic stenosis, subendocardial fibroelastosis and trabecular hypertrophy, and other miscellaneous cardiac abnormalities (Uzal et al. 2026). A cardiac examination protocol was established to standardize the gross and microscopic examination of the heart in cases of SD, particularly those of suspect cardiac origin (Diab et al. 2017; Uzal et al. 2026). The protocol includes examination of 11 regions targeting major structures, including the sinoatrial and atrioventricular nodes (Diab et al. 2017; Uzal et al. 2026). Minimal to mild cardiac lesions such as interstitial infiltrates of lymphocytes, plasma cells, and macrophages; fibrosis; and hypercontraction bands, are usually encountered, but the limited distribution and/or severity of such findings, and the fact that similar changes are frequently encountered in healthy control horses, make it difficult to attribute them some significance (Uzal et al. 2026).

Here we describe a case of EASD in a Thoroughbred racehorse in which the histology protocol was used to map significant cardiac lesions of unknown cause, which were presumptively secondary to an infected castration wound.

## Case presentation

The carcass of a 3-year-old Thoroughbred gelding was submitted to the San Bernardino Laboratory of the California Animal Health and Food Safety Laboratory System (CAHFS) for postmortem examination. The horse collapsed and died on a dirt track a few minutes following an early morning workout.

**Fig. 1:**
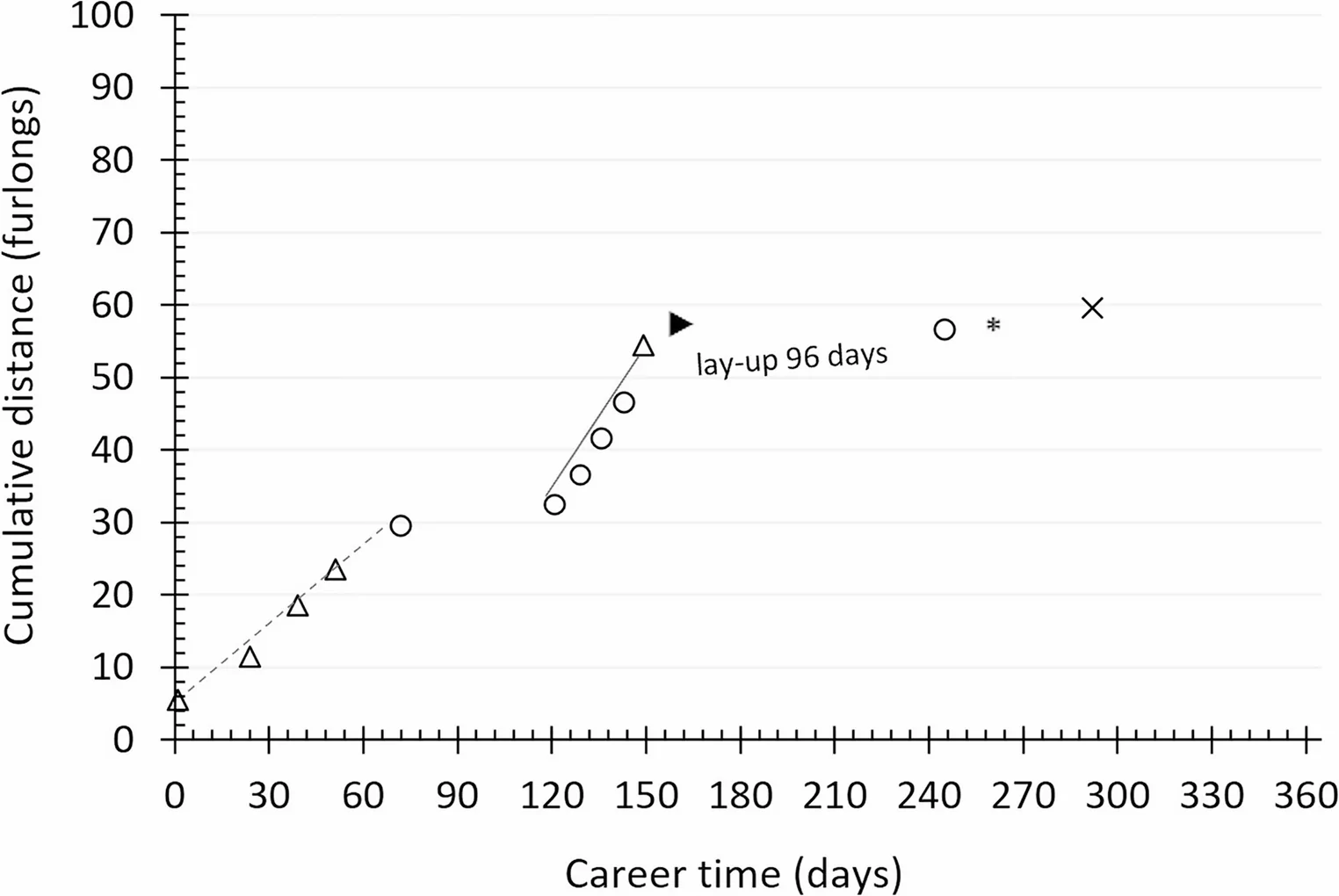
Cumulative distance in furlongs, including races and workouts over a 292-day period, and relevant medical interventions. The horse completed 26 furlongs in workouts within one month. After a 96-day lay-up, the horse completed one workout, followed by the workout during which it died. (∆) races, (○) workouts, (x) death, (―) 26 furlongs/month, (---) 7 furlongs/month, (*) castration, (►) arthroscopic surgery

Over a span of 292 days, this horse had a history of 6 races, 5 of which occurred over a 72-day span, accumulating 29 furlongs. Additionally, the horse completed 4 workouts over a 23-day period, accumulating 46 furlongs. The horse underwent arthroscopic surgery on the right front fetlock, followed by a 96-day lay-up. A workout was then completed, followed by castration. Twelve and 15 days post-castration, antibiotics (EXCEDE^®^ [Ceftiofur Crystalline Free Acid]) were administered. Due to apparent infection, swelling, and lack of drainage at the surgical site, the area was opened aseptically 21 days after castration, and enrofloxacin was administered for five consecutive days. On the day of the horse’s death, the animal had accumulated a total of 54 furlongs from both races and recorded intensive workouts (Fig. 1).

A full necropsy, including a full cardiac examination as previously described (Diab et al. 2017; Uzal et al. 2026), was performed approximately 7 h after death. The carcass was fresh and had good nutritional condition. The scrotum was moderately swollen, edematous, and had an open, approximately 8 × 3 cm surgical wound with pockets of pus. The adjacent subcutis of the inguinal area and caudal abdomen, as well as the spermatic cord stump, were unremarkable. Both lungs were non-collapsed, swollen, and had rib impressions on the dorsal and lateral surfaces of the caudal lobes. Abundant stable foam was present in the lumen of the trachea and lower airways. There were multifocal faint ecchymoses on the epicardial and endocardial surfaces but the heart was otherwise unremarkable grossly. The spleen was congested and enlarged up to 4 times its normal size. In the non-glandular portion of the stomach, there were multifocal, mild ulcers. Both forelimbs distal to the radio-carpal joints were examined and there was mild degenerative joint disease in the carpal bones, proximal sesamoid bones, and the condyles of the third metacarpal bone, which was considered incidental.

Samples of lungs, liver, kidneys, lymph node, spleen, thyroid glands, stomach, esophagus, scrotum (surgical site with adjacent subcutis and muscle), skeletal muscle, brain, hypophysis, small intestine, large intestine, trachea, and heart (11 regions as previously described [Diab et al. 2017; Uzal et al. 2026]; Supplementary Fig. 1), were collected and fixed in 10% neutral-buffered formalin for 24 h. All the tissues were routinely processed for the production of 4 μm-thick, H&E-stained sections. Selected sections of heart were stained with Gram, Steiner, Picrosirius red, and Masson’s trichrome stains.

**Fig. 2:**
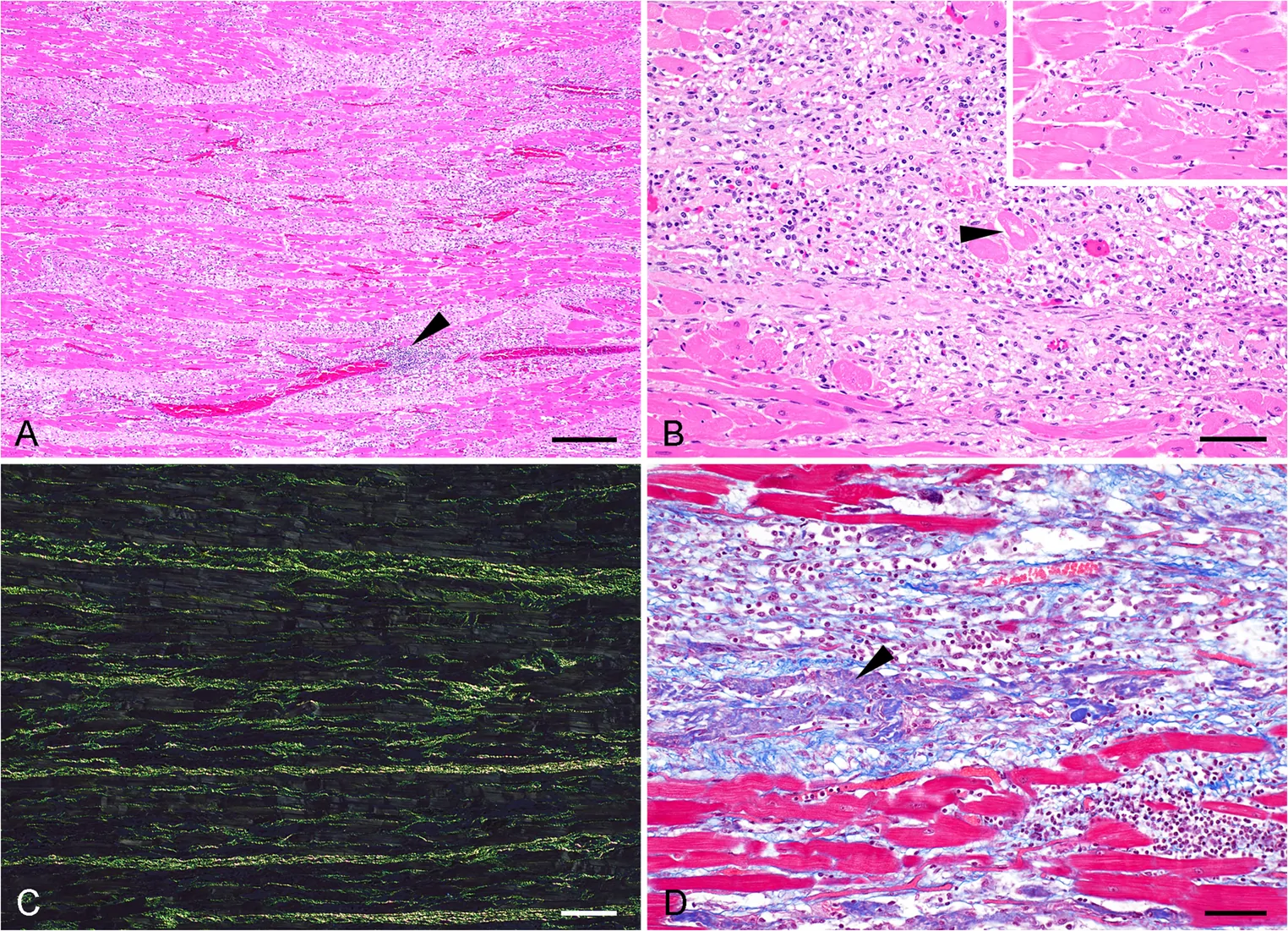
Cardiac microscopic lesions in a Thoroughbred racehorse that died suddenly after a workout. (**A**,** C**,** D**)**.** Region 10, interventricular septum. (**B**). Region 11, left ventricular outflow tract. (**A**) Multifocal to coalescing areas of cardiomyocyte loss, fibroplasia, and focal perivascular aggregate of lymphocytes and plasma cells (arrowhead). H&E. Bar = 400 μm (**B**) Focally extensive fibroplasia with cardiomyocyte degeneration and necrosis (arrowhead) and scattered lymphocytes, plasma cells and macrophages. H&E. Bar = 150 μm. Inset: Higher magnification of the necrotic and degenerated cardiomyocytes. (**C**) Diffuse severe myocardial fibrosis. Picrosirius red stain, polarized light. Bar = 200 μm. (**D**) Fibrosis with numerous lymphocytes, macrophages, and plasma cells and necrotic cardiomyocytes (arrowhead). Masson’s trichrome stain. Bar = 150 μm

Within the heart (Table 1), affecting several regions but being particularly severe and extensive in the myocardium of the left and right ventricular free walls and interventricular septum, there were different combinations of focally extensive, multifocal-to-coalescing or diffuse cardiomyocyte degeneration and necrosis, interstitial infiltrates of lymphocytes and plasma cells, and fibroplasia (Fig. 2A-D). No microorganisms were observed on H&E or special stains. All lung sections had diffuse alveolar and interstitial edema, and severe congestion. The spleen was severely congested.

**Table 1:** Cardiac microscopic examination results in a Thoroughbred racehorse

Region^a^	Distribution^b^		% of affected section^c^	Cardiomyocyte degeneration/ necrosis severity^d^	Lymphoplasmacytic inflammation, location	Fibroplasia	Hemorrhage
1	FE	40		++	Yes, interstitial and perivascular	Yes	No
2	FE to D	90		+++	Yes, interstitial and perivascular	Yes	No
3	MF	5		+	Yes, interstitial and perivascular	No	No
4	F	5		-	Yes, interstitial	No	No
5	MF	5		-	Yes, interstitial and perivascular	No	Yes
6	MF	20		+	Yes, interstitial	Yes	No
7	MF	40		++	Yes, interstitial and perivascular	Yes	No
8	MFC	70		+++	Yes, interstitial and perivascular	Yes	No
9	MFC	20		++	Yes, interstitial and perivascular	Yes	No
10	MFC to D	90		+++	Yes, interstitial and perivascular	Yes	No
11	MFC	70		+++	Yes, interstitial and perivascular	Yes	No

^a^Regions examined and main location of lesions within the sections (Supplementary Fig. 1 [Diab et al. 2017; Uzal et al. 2026]): #1, right ventricular free wall, with the right atrial wall, the right coronary artery, and the parietal cusp of the tricuspid valve (lesions in myocardium of the right ventricular free wall); #2, pulmonary artery semilunar valve with the right ventricular outflow tract and the pulmonary artery (lesions in myocardium of right ventricular outflow tract); #3, right atrial appendage (lesions in myocardium); #4, sinoatrial node region (lesions in myocardium away from the node); #5, left atrial appendage (lesions in myocardium); #6, left ventricular free wall, left atrial wall, left coronary artery, and the parietal cusp of the left atrioventricular valve (lesion in myocardium of the left ventricular free wall and left atrial wall); #7, left ventricular papillary muscle 1 of 2 (lesions in myocardium); #8, left ventricular papillary muscle 2 of 2 (lesions in myocardium); #9, atrioventricular node with the orifice of the coronary sinus, right atrium, and right ventricle (lesions in myocardium away from the node); #10, interventricular septum with one endocardial surface (lesions in myocardium); and #11, aortic semilunar valve, aorta, and left ventricular outflow tract (lesions in myocardium of left ventricular outflow tract)

^b^Distribution of all lesions combined within the examined section: FE, focally extensive; D, diffuse; F, focal; MF, multifocal; MFC, multifocal to coalescing

^c^% affected section: Percentage of section affected by all lesions combined estimated subjectively

^d^Severity of cardiomyocyte degeneration and necrosis: -, Absent; +, Mild; ++, Moderate; +++, Severe

In the scrotum, there was epidermal ulceration with superficial infiltrates of neutrophils and macrophages, necrosis, cell debris, granulation tissue with microthrombi in the capillaries, and myriad cocci. In the kidneys, there were multifocal interstitial infiltrates of macrophages, lymphocytes, and plasma cells admixed with karyorrhectic material (necrosis).

Additional ancillary tests were performed following CAHFS standard operating procedures and as per protocols for California Horse Racing Board necropsy cases, and included: aerobic bacterial culture on liver, lung, scrotum, heart, and kidney; anaerobic bacterial culture on scrotum; *Salmonella* spp. RT-qPCR screen from liver; *Leptospira* spp. qPCR and immunohistochemistry (IHC; multivalent fluorescent antibody conjugate, National Veterinary Services Laboratory LEP-FAC; 1:3,000) on kidney; West Nile virus IHC (rabbit polyclonal, Bioreliance 7H2, 1:400) on kidney and heart; fecal flotation; alkaloid screen (including anabasine, atropine, coniine, deltaline, nicotine, scopolamine, sparteine, and taxus) on gastrointestinal contents; anticoagulant rodenticide screen (including brodifacoum, bromadiolone, chlorophacinone, coumachlor, difethialone, diphacinone, warfarin, and difenacoum) on liver; ionophores screen (including lasalocid, monensin, narasin, salinomycin) on liver; oleander glycosides analysis on liver; extended heavy metal screen (including arsenic, cadmium, cobalt, copper, iron, lead, manganese, mercury, molybdenum, selenium, zinc) on liver; vitamin E determination on liver; and gas chromatography mass spectrometry (GCMS) and liquid chromatography mass spectrometry (LCMS) on liver. In addition, qPCR for *Borrelia burgdorferi* was performed on heart at the Animal Health Diagnostic Center, College of Veterinary Medicine, Cornell University, Ithaca, NY.

A pure culture of moderate numbers of *Streptococcus equi* ssp. *zooepidemicus* was obtained from the scrotum. Moderate numbers of *Carnobacterium divergens* and mixed flora were isolated from the kidney. Phenylbutazone was detected in the LCMS and GCMS screens, and enrofloxacin was detected in the LCMS screen. The rest of the tests and screens were negative or within expected ranges.

### Discussion and conclusion

The cardiac examination protocol was used in this case to identify and map pre-existing lesions in the heart. These lesions were very severe and extensive in some sections, and were therefore considered as very likely contributors to death. There are two types of cardiac lesions identified in racehorses with EASD: those interpreted as contributors to death and those interpreted as incidental (DeLay 2017; Lyle et al. 2011; Molesan et al. 2019). This distinction is usually made by the pathologist(s) performing the necropsy, and the criteria used may vary, but generally include lesion location (e.g., affecting the conduction system) and distribution (e.g., percentage of affected tissue within specific sections and number of sections affected) (Uzal et al. 2026). In our case, most heart sections had lesions, but severity varied widely, emphasizing the utility of examining several regions to identify those most affected and to estimate overall extension within the heart. Even though the sections of atrioventricular and sinoatrial nodes were just mildly to moderately affected in our horse, the fact there were severe and extensive lesions in the ventricular free walls and septum supports the idea that cardiac malfunction and/or arrythmia may have occurred. In fact, the extensive intramural Purkinje network forms a substantial component of the cardiac conduction system, which may have been impacted in this case due to the widespread myocardial involvement. As such, the mechanism of death was likely cardiogenic shock with increased hydrostatic pressure, fluid leakage from the pulmonary capillaries, and pulmonary edema.

No nutritional deficiencies, cardiotoxins or infectious agents contributing to the cardiac lesions were identified. The horse had been castrated a month prior to death, and we found an open castration wound with superficial, localized bacterial infection from which *Streptococcus equi* subsp. *zooepidemicus* was isolated. Our main hypothesis is that an embolic shedding from this wound could have impacted different areas of the heart, and perhaps the kidney, eventually causing the observed cardiac lesions and malfunction (Robert et al. 2017). However, no bacteria were visualized histologically on H&E, Gram or Steiner stains, or cultured from the heart, and similar embolic lesions in other organs, peritonitis or general signs of sepsis were not observed. Alternatively, a persistent localized bacterial infection in the scrotum could have induced an immune-mediated reaction targeting the heart without bacterial translocation (Sikder et al. 2018). In fact, there are some *Streptococcus* spp. as those within group G that can cause rheumatic heart disease (Sikder et al. 2018). In those cases, the streptococcal M protein induces autoreactive T cells via molecular mimicry that can cross-react with host tissue proteins, triggering an inflammatory response in the heart (Sikder et al. 2018). IHC for major histocompatibility complex, components of the complement system, or immunoglobulins have been performed in cases of inflammatory neuromuscular disease in humans (Nelke et al. 2024). This approach has never been validated or explored in equine EASD cases, but future retrospective studies could be performed to test those markers. The cause of the renal lesions was not determined, with the bacteria isolated from the kidney interpreted as contaminants and/or incidental; however, a similar pathogenesis as that suggested for the cardiac lesions (i.e., embolic bacterial shower or immune-mediated reaction) is possible for the lesions in the kidneys too.

Prolonged periods of inactivity in racehorses increase the risk of catastrophic musculoskeletal injuries when animals are returned to high intensity activity (Stover et al. 1992; Carrier et al. 1998). An association between a recent history of > 60 days lay-up and EASD has also been suggested (Bennet and Parkin 2022), but the mechanism(s) involved are poorly understood. In this line, this horse had a history of intense racing and training over a span of 292 days, until approximately 4 months before death, when a > 3-month lay-up occurred after an arthroscopic surgery, followed by a single-timed workout completed before death. Longer race length is considered a fatality risk factor in racehorses (Boden et al. 2006); however, in our case, as significant pre-existing cardiac pathology was observed, this level of activity is unlikely to have had a primary role in the development of the lesions. It is, however, unknown whether persistent bouts of intense exercise combined with other factors such as the scrotal infection could have contributed to cardiac damage over time, but the role of exercise in development of myocardial fibrosis and other cardiac lesions is not clear overall (Uzal et al. 2026).

Toxic substances such as oleander glycosides and other cardiotoxic alkaloids part of a broader screen were ruled out as contributors to the cardiac lesions. We tested for other toxic substances to the best of our technical means, but it remains possible that different compounds not included in the tests performed escaped detection or that the causative substance was not present anymore at the moment of testing, since the cardiac lesions were somewhat chronic. However, we consider this unlikely, as the potential sources of exposure (i.e., feed, water) were shared by other horses and similar cases would have been expected within an analogous time frame. Likewise, adequate hepatic concentrations of vitamin E and selenium are not compatible with nutritional myopathy. The compounds detected in the LCMS and GCMS screens were interpreted as therapeutic and unrelated to death.

Other potential causes include genetic defects, which we did not test for in this horse. An example of the latter is arrhythmogenic right ventricular cardiomyopathy (Freel et al. 2010); however, lesions in this disease tend to be restricted to the right ventricle, and consist of infiltrates of fibro-fatty tissue, which is different to the combination of cardiomyocyte death, fibroplasia, and inflammation observed in this horse.

Prevention of fatalities such as this one is critically important to ensure horse welfare. As the cause of the cardiac lesions in this case was not completely elucidated, it is only speculative whether some pre-mortem clinical management could have prevented death. However, routine medical exams with emphasis on the respiratory and cardiovascular systems could have identified some abnormalities, and, if the cause of the cardiac lesions was indeed the infected scrotal wound, proper perioperative management post-castration, including adequate resting, would have prevented the fatal outcome.

In this case, the cardiac examination protocol was a useful tool to map cardiac lesions and estimate their role in this horse’s death. The case work-up can serve as an example for pathologists and regulators working with similar horses in the future, and emphasizes the necessity of a systematic approach to racehorse necropsies.

## Supplementary Information

Below is the link to the electronic supplementary material.

Supplementary File 1 (DOCX 356 KB)

## Data Availability

All data generated or analyzed during this study are included in this published article.
