# Supplementary material for: Case report: exercise-associated sudden death in a Thoroughbred racehorse with cardiac lesions
Source: Vet Res Commun. 2026 Jul 4;50(5):439. doi: 10.1007/s11259-026-11384-0 (PMC13332897; doi:10.1007/s11259-026-11384-0)
Supplement: Supplementary file 1 — (DOCX 356 KB) [file 11259_2026_11384_MOESM1_ESM.docx]

**Acevedo et al: Case report: Exercise-associated sudden death in a Thoroughbred racehorse with cardiac lesions**

**Supplementary materials**


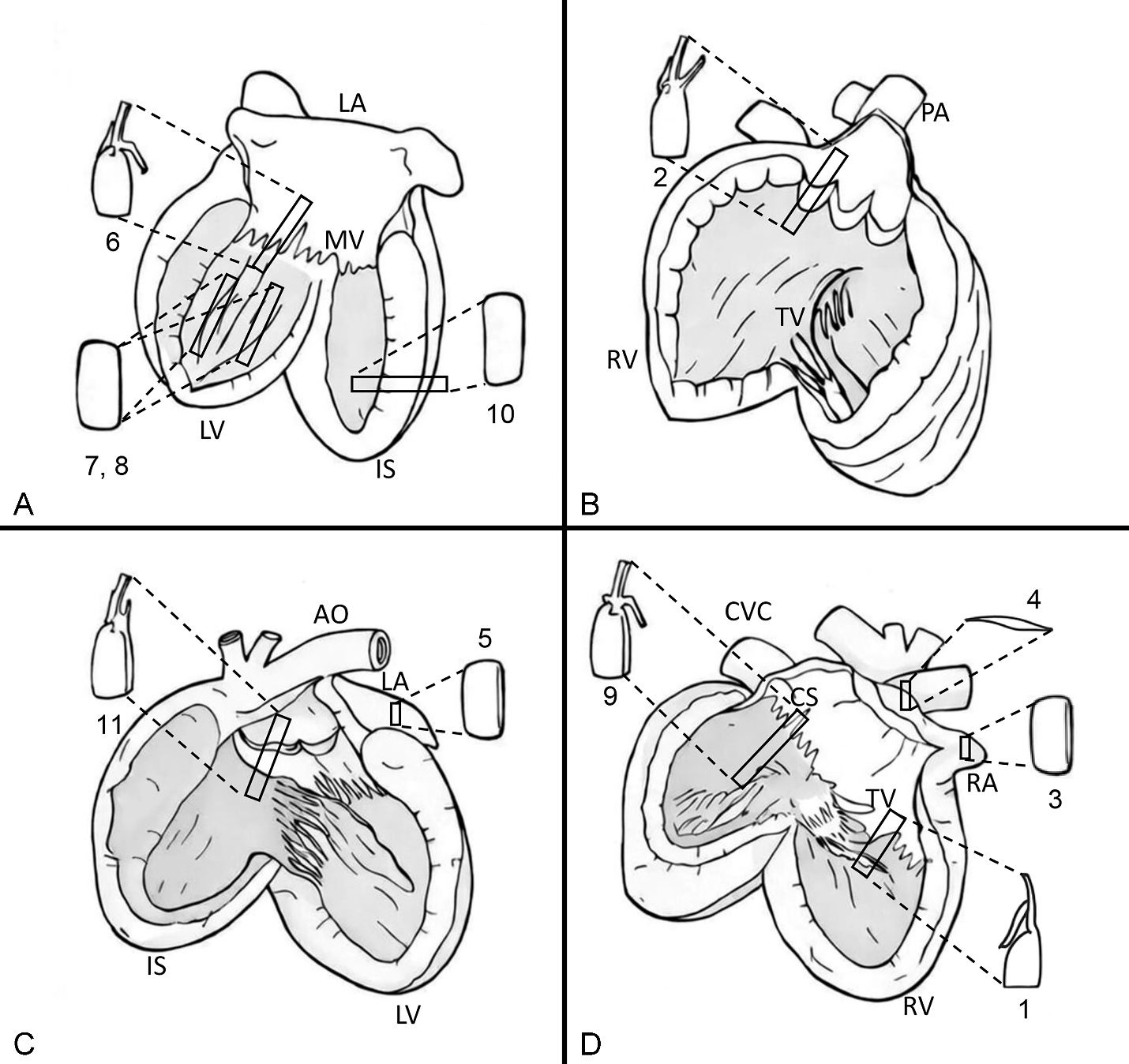


**Supplementary Figure 1***. Diagrams with location of the 11 regions of interest for histologic examination of the equine heart in cases of exercise-associated sudden cardiac death. A. Regions 6 (left ventricular free wall, left atrial wall, left coronary artery, and the parietal cusp of the left atrioventricular valve), 7 (left ventricular papillary muscle 1 of 2), 8 (left ventricular papillary muscle 2 of 2), 10 (interventricular septum with one endocardial surface). B. Region 2 (pulmonary artery semilunar valve with the right ventricular outflow tract and the pulmonary artery). C. Regions 5 (left atrial appendage), 11 (aortic semilunar valve, aorta, and left ventricular outflow tract). D. Regions 1 (right ventricular free wall, with the right atrial wall, the right coronary artery, and the parietal cusp of the tricuspid valve), 3 (right atrial appendage), 4 (sinoatrial node region; the sinoatrial node is located in the subepicardial region of the superior border of the terminal sulcus, at the junction of the cranial vena cava and the right atrium), 9 (atrioventricular node with the orifice of the coronary sinus, right atrium, and right ventricle; the atrioventricular node is located in the subendocardial region of the right atrial wall, cranial to the orifice of the coronary sinus and slightly above the junction of the septal cusp of the tricuspid valve). AO, aorta; CS, coronary sinus; CVC, cranial vena cava; IS, interventricular septum; LA, left atrium; LV, left ventricle; MV, mitral valve; PA, pulmonary artery; RA, right atrium; RV, right ventricle; TV, tricuspid valve.

***Adapted from:**

1. Fox PR (ed.), Bishop SP. Canine and Feline Cardiology, 1st ed., Churchill Livingstone, 1988

2. Robinson WF, Maxie MG. The Cardiovascular System. In Jubb KVF, Kennedy PC, Palmer N, eds. Pathology of Domestic Animals. 4th ed. Vol. 3. Academic Press, Inc., 1993:1-100
